# Attention in our digital ecosystem: The five interactive components that drive media multitasking

**DOI:** 10.3758/s13423-025-02722-5

**Published:** 2025-06-27

**Authors:** Allison C. Drody, Effie J. Pereira, Daniel Smilek

**Affiliations:** 1 Department of Psychology, University of Waterloo, PAS Building, 200 University Avenue West, Waterloo, ON N2L 3G1 Canada; 2 Department of Psychology, Queen’s University, Kingston, ON Canada; 3 Centre for Neuroscience Studies, Queen’s University, Kingston, ON Canada

**Keywords:** Media multitasking, Attention, Interactive model, Dual-tasking, Digital media

## Abstract

Advances in the portability of digital media devices and the sophistication of digital media platforms have dramatically changed how we engage with media content and the degree to which we media multitask in everyday life. While a large body of research on dual-tasking has laid the foundation for our understanding of media multitasking, research has only begun to explore the voluntary and dynamic factors that account for how we simultaneously engage with media-based sources. Here, we consider recent studies assessing non-required instances of media multitasking and propose that this behaviour can be best understood through an interactive model that operates along a core temporal dimension, varying from stable to transient, and contains cognitive architectural, dispositional, metacognitive, task-valuation, and environmental components that drive media multitasking behaviours. By detailing how each of these five components heightens or reduces an individual’s likelihood of engaging in media multitasking, we showcase the utility of the interactive model in guiding future research and accounting for the dynamic ways this behaviour is embedded within our real and digital lives.

**Supplementary Information:**

The online version contains supplementary material available at 10.3758/s13423-025-02722-5.

## Introduction

Attention is one of the most valuable commodities we possess within our everyday lives. In addition to controlling what we select in our environment and how long we sustain our focus on it, this fundamental neurocognitive process affords us the ability to divide our cognitive resources as needed (Broadbent, 1958; Cherry, 1953; James, 1890; Kahneman, 1973; Ribot, 1890; Treisman, 1960). Across daily life, we make ample use of this ability by simultaneously engaging in multiple tasks through dual-tasking, such as working while talking to a co-worker, driving while planning a to-do list, or watching TV while cooking. Although dual-tasking behaviours are universal to our everyday experience, the evolution of our technological society, with the increased availability of media devices and greater access to media options, has altered what dual-tasking looks like across our everyday digital experience. Now, working might be done while attending to text conversations, driving could be paired with listening to a podcast, and watching TV might be accompanied by playing a game on one’s smartphone. These examples provide clear illustrations of *media multitasking*, a form of dual-tasking behaviour that is embedded in digital spheres.

Although our understanding of media multitasking has been borne from a rich body of work on dual-tasking, there are two core characteristics that distinguish media multitasking from dual-tasking. That is, media multitasking might entail simultaneous engagement with at least two sources, but in addition it also involves (1) media-based sources that serve an entertainment and/or social role, and (2) non-required engagement with the media-based source, which provides for a free, willful, and dynamic selection of said source at any given moment in time. In this way, media multitasking brings to the forefront media-specific characteristics and discretional opt-in dynamics that distinguish this behaviour from how dual-tasking has been studied in the past, necessitating a more comprehensive understanding of the factors that drive media multitasking.

The current paper addresses a critical and emerging question within the field of cognitive science: What are the principal levels of influence that give rise to media multitasking? In this article, we first discuss prior research on dual-tasking as a means of defining the core construct, outcomes, and models that this body of work has produced. Then, we establish how media multitasking distinguishes itself from dual-tasking across core characteristics of media centricity and non-required engagement, and detail current methods of measuring media multitasking that prioritize these features. After underscoring this knowledge gap, we bridge diverse fields of work to present a strong argument for why media multitasking is best encapsulated by an interactive model that operates under five distinct components – cognitive architectural, dispositional, metacognitive, task-valuation, and environmental – that work together to determine whether an individual will media multitask at any given moment in time. Finally, we illustrate how this novel conceptualization provides a clear path forward for future research that can account for the dynamic ways that we engage in media multitasking within the laboratory and within our real and digital worlds.

### A look back at dual-tasking

Over the decades, the cognitive and neural mechanisms that enable or limit our ability to dual-task have been examined in multiple ways, with conceptual shifts in the boundaries and classification of this behaviour occurring to accommodate these various perspectives. One class of dual-task research paradigms involves having participants complete two tasks that unfold *simultaneously in time* (Baddeley & Hitch, 1974; Broadbent, 1958; Fisk et al., 1986; Kahneman, 1973; Karatekin et al., 2004; Mcdowell et al., 1997; Spelke et al., 1976; Strayer et al., 2022). For example, in their investigations of working memory, Baddeley and Hitch (1974; see also Baddeley, 1998, p. 50) used a dual-task technique that involved having participants simultaneously perform two tasks that both ostensibly required working memory capacity (e.g., digit span and a reasoning task). Similar paradigms were used in early studies of divided attention, wherein participants were required to read a story and simultaneously listen to a different story (Mowbray, 1953) or write down dictated words (Spelke et al., 1976; see Kahneman, 1973, for other examples). Also related is work from Broadbent (1958) that assessed participants’ ability to extract information from multiple channels at once (e.g., different streams of information simultaneously presented to each ear). The consequences of dual-tasking have also been explored in applied contexts, as exemplified by the work of Strayer and colleagues (e.g., Strayer et al., 2022), in which participants were required to complete cognitively demanding tasks (e.g., counting backwards) while driving an automobile in a simulator.

Another class of dual-task research paradigms includes studies in which participants are required to complete two tasks *in rapid succession* (e.g., Arrington & Logan, 2004; Dreisbach et al., 2007; Luria & Meiran, 2003; Mayr & Bell, 2006; Monsell, 2003; Pashler, 1994; Shapiro et al., 1997; Telford, 1931; Tombu & Jolicoeur, 2003). One specific example is the psychological refractory period paradigm (Pashler, 1994; Telford, 1931; Tombu & Jolicoeur, 2003), in which two stimuli are presented and responded to in rapid succession, with varying intervals between their onsets. Similarly, the attentional blink paradigm requires participants to identify two successively presented stimuli in a stream of rapidly presented stimuli (Shapiro et al., 1997). Also relevant is the task-switching paradigm, in which participants perform the same task in succession or switch from one task to another (Monsell, 2003), allowing researchers to observe the costs of rapidly switching between tasks.

Studies using these various dual-task research paradigms have led to several broad findings and spawned a variety of theoretical accounts. One such general finding is that performing more than one task simultaneously or in close succession can lead to impaired performance on one or both tasks (Arrington & Logan, 2004; Borst et al., 2010; Jansen et al., 2016; Luck, 1998; Nijboer et al., 2014; Strobach et al., 2015; Wylie & Allport, 2000). For instance, simultaneously completing a virtual driving task and an auditory memory task leads to worse performance compared to when the two tasks are completed separately (Jansen et al., 2016). Similarly, rapidly switching between tasks, such as different versions of a Stroop task (e.g., word reading vs. colour naming) results in slower response times compared to repeatedly performing the same version of the task instead (Wylie & Allport, 2000). However, whether, or to what degree, impairments occur appears to depend on various task factors, including the temporal interval of the tasks, the difficulty of the tasks, and the similarity of the tasks in terms of their perceptual, cognitive, and response demands (Adler & Benbunan-Fich, 2015; Altmann, 2004; Bier et al., 2017; Göthe et al., 2016; Hwang & Jeong, 2018; Schaeffner et al., 2018; Strobach et al., 2015). For instance, switch costs are generally lower when the temporal intervals between tasks are longer (e.g., when there is a greater interval between the onset of two target stimuli requiring different responses; Strobach et al., 2015); task performance is often much worse when increasing task difficulty (e.g., increasing target speed on a visual tracking task while simultaneously completing a digit span task; Bier et al., 2017); and greater performance detriments are seen when completing two tasks within the same modality (e.g., a visual symbol copying task while monitoring for visual flashes of light) versus when the tasks have minimal overlap in modality (e.g., a visual symbol copying task while monitoring for auditory cues; Hwang & Jeong, 2018).

To explain these patterns of effects, various cognitive models have been proposed to account for dual-tasking behaviours, primarily from a resource-driven framework. While originally intended for understanding the neural underpinnings of dual-tasking, these models can also offer some insight into why individuals engage in this behaviour. For example, models that include bottlenecks in processing (Broadbent, 1958; Pashler, 1994), limited capacity-sharing mechanisms (Kahneman, 1973; Navon & Gopher, 1979; Tombu & Jolicoeur, 2003), multiple resource pools (Baddeley, 1998; Navon & Gopher, 1979; Wickens, 1984), supervisory attentional systems (Norman & Shallice, 1986), or multiple task-threading processes (i.e., threaded cognition; Salvucci & Taatgen, 2008) all conceptualize dual-tasking as being driven by a fixed pool of cognitive resources that individuals have available at any given moment in time. If a given task does not utilize all of these resources, the additional resources that are left in reserve can be directed towards other tasks, leading to dual-tasking behaviours. From these perspectives, dual-tasking is said to be driven by stable components within individuals that seek to resolve an underutilization of resources.

In summary, the prior body of work on dual-tasking has solidified this behaviour as being characterized by the simultaneous engagement in two tasks that an individual is required to complete. Much of this dual-tasking work has focused on observing performance during various task combinations in order to advance cognitive theory. The results have revealed that dual-task-related performance detriments often depend on the degree to which the tasks rely on similar cognitive resources.

### A look forward to media multitasking

Given the large body of work on dual-tasking, the first important question is what differentiates research on media multitasking from previous research on dual-tasking? The answer to this question can be found in the way dual-tasking and media multitasking are typically defined. As we will see, the definition of media multitasking gives rise to research questions that go beyond those that have been extensively explored in the dual-tasking literature.

In terms of definitional distinctions, media multitasking can be approached from the family resemblances perspective (Wittgenstein, 1953), which has been fruitfully applied to related cognitive concepts such as attention (Moray, 1969) and mind wandering (Seli et al., 2018). Briefly, the family resemblance perspective construes concepts as having fuzzy boundaries, with category membership driven by a concept’s degree of prototypicality along a series of relevant characteristics. With this view, a prototypical case of media multitasking would involve the two unique core characteristics of (1) media centrality and (2) non-required engagement. *Media centrality* speaks to the simultaneous engagement in two tasks or two sources of information, of which at least one is media-based (e.g., Beuckels et al., 2021; Lang & Chrzan, 2015; Matthews et al., 2022; Van der Schuur et al., 2015). Although the term *media-based source* itself can be defined in various ways, as per the current literature on media multitasking, we can take this to primarily refer to modern digital mass media and communication technologies that often serve an entertainment or a social purpose, which includes television, video, social media, video-based communication, and texting (e.g., Baumgartner et al., 2017; Beuckels et al., 2021; Kononova & Yuan, 2017; Wang & Tchernev, 2012). *Non-required engagement* refers to engagement in media-based content, such that media multitasking is not forced on by the situation or mandated by an authority (e.g., an experimenter or task instructions; Brasel & Gips, 2017; Lopez & Orr, 2022; Ralph et al., 2020). This non-requirement would allow for the flexible and dynamic selection of media sources at any given time, which could reflect an individual’s deliberate decision to media multitask or spontaneous selection of media tasks based on in-the-moment cues like smartphone notifications (Bartoli & Benedetto, 2022; although, see Wiradhany et al., 2024).

These defining features lead to different research objectives across dual-tasking and media multitasking research. Concerning *media centrality*, prototypical research on dual-tasking has primarily used secondary tasks designed to test specific theoretical questions about the mechanisms underlying divided attention, such as by pairing a letter response task with an auditory tone (Pashler, 1994) or completing word- and colour-naming tasks in rapid succession (Wylie & Allport, 2000). This approach is useful for examining performance decrements when cognitive resources are shared across tasks, but they do so by decentering any motivational components that would result in individuals specifically multitasking with media, such as a desire for social connection (Chang, 2017; Kononova & Chiang, 2015; Kononova & Yuan, 2017) or a fear of missing out/anxiety (González et al., 2025; Shane-Simpson & Bakken, 2024; Wu et al., 2025; Zhao, 2023). Moreover, dual-tasking does not account for the influence of factors external to the individual, such as design features of digital platforms which aim to attract and maintain our attention (Bhargava & Velasquez, 2021; Bruineberg, 2023; Flayelle et al., 2023). Understanding these components is at the forefront of most media multitasking research. Regarding *non-required engagement*, most studies of dual-tasking pre-select the secondary task for participants, such as by having participants respond to different stimuli within the context of the same cognitive task or by having participants engage in a secondary task that differs from their primary task (e.g., watching a pre-selected video while reading an article; Lee et al., 2012; Lin et al., 2009, 2011). Additionally, they often provide participants with explicit instructions on how to engage with the assigned tasks (e.g., dividing attention across both tasks equally or prioritizing one task over another; Lee et al., 2012). These forced requirements may cause participants to rigidly divide cognitive resources based on the parameters of the task in order to favour optimal performance or follow researcher instructions. As a result, dual-tasking may capture behaviours that are largely tied to preparatory or planned responses. In contrast, studying how individuals multitask with media when such behaviour is not explicitly required of them is a key focus of media multitasking research.

In sum, multitasking situations in which media streams are paramount (media centrality) and individuals have choice in whether they engage in this behaviour (non-required engagement) characterize prototypical cases of media multitasking but not classically studied dual-tasking. These key defining features have led to a shift away from how cognitive information-processing models can account for the performance costs of multitasking – a primary focus of dual-tasking research – towards how and why individuals combine specific media-based activities – a key focus of media multitasking research. Exploring this latter issue is crucial, as media multitasking has been associated with impaired performance across a variety of contexts (e.g., Demirbilek & Talan, 2018; Drody et al., 2022, 2023; Lau, 2017; Lopez & Orr, 2022; Ralph et al., 2020, 2021) and individuals often media multitask despite being aware of these potential costs (Calderwood et al., 2016; Ralph et al., 2020). There is therefore a need to better understand what drives this behaviour.

### How do we capture media multitasking?

Having provided a definitional framework for media multitasking and having distinguished it from prior cognitive studies of dual-tasking, we can consider how this distinct behaviour has been measured in the extant literature. Most current studies examining media multitasking have taken one of three approaches. The first involves assessing media multitasking via questionnaires (e.g., Baumgartner et al., 2017; Ophir et al., 2009), the second focuses on observing and probing instances of media multitasking in real-world settings (e.g., Moreno et al., 2012; Ragan et al., 2014; Rosen et al., 2013), and the third involves offering participants the opportunity to media multitask in the context of laboratory tasks (e.g., Brasel & Gips, 2017; Ralph et al., 2020). Below we consider each of these in turn.

#### Questionnaire-based measures

Assessments of media multitasking via questionnaire-based measures began with the foundational work of Ophir and colleagues (2009), who developed the Media Multitasking Index (MMI) to capture how often per week an individual engages in different media combinations. The researchers used this measure to explore variability in media multitasking by asking whether chronic media multitasking was associated with improvements or impairments in cognitive control (e.g., attending to important information, ignoring irrelevant information). Scores on the MMI are often used to distinguish between heavy and light media multitaskers and to observe relationships between increased tendencies towards media multitasking and various traits and outcomes, such as greater impulsivity (Minear et al., 2013; Müller et al., 2021; Sanbonmatsu et al., 2013), higher sensation seeking (Chang, 2017; Duff et al., 2014; Sanbonmatsu et al., 2013), and poorer working memory (Murphy & Creux, 2021; Ralph & Smilek, 2017; Uncapher et al., 2016). Other measures of media multitasking have since been developed and used to explore similar questions and topics, albeit with an updated focus on modern forms of media. Examples include an alternate version of the MMI (i.e., MMI-2; Ralph et al., 2015; Ralph & Smilek, 2017), as well as the Short Media Multitasking Measure (i.e., MMM-S; Baumgartner et al., 2017).

A clear benefit of these questionnaires is that they undoubtedly capture the two core characteristics of media multitasking by specifically indexing combinations of multiple media sources and assessing individuals’ tendencies to combine these activities in daily life. However, one drawback of this research has been that studies using questionnaire-based assessments have suffered from a lack of consistency in findings across studies. For instance, some studies have found relations between high MMI scores and poor performance on tasks requiring cognitive control and sustained attention (Baumgartner et al., 2014; Cain et al., 2016; Ophir et al., 2009), whereas others do not (Ralph & Smilek, 2017; Ralph et al., 2015; Rogobete et al., 2020; Seddon et al., 2018). Recent meta-analyses have revealed that studies using these measures often suffer from small effect sizes and display a high degree of heterogeneity (Kong et al., 2023; Wiradhany & Koerts, 2021; Wiradhany & Nieuwenstein, 2017), highlighting the notion that questionnaire-based assessments may capture an individual’s general aggregate tendency to combine different forms of media, but they do so at the expense of accounting for any temporal factors that may impact this behaviour. For example, two individuals who on average media multitask the same amount with the same media sources may have different outcomes due to differences in (1) how they disperse their media multitasking (e.g., dividing attention equally and consistently across the tasks vs. intermittently shifting attention from task to task), (2) why they media multitask (e.g., information-seeking vs. entertainment), and/or (3) where they media multitask (e.g., studying in class vs. relaxing at home). Thus, while questionnaire-based measures may adequately capture the two core characteristics of media centrality and non-required engagement, they fail to account for specificity within each.

#### Observational approaches

Measuring media multitasking through observation has typically involved having researchers monitor for (Ragan et al., 2014; Rosen et al., 2013; Voorveld & Viswanathan, 2015) or probe (Wammes et al., 2019) instances of this behaviour in the real world. For instance, researchers have observed and recorded instances of media multitasking within learning environments, such as during 80-min classes (Wammes et al., 2019) or 3-h lectures (Ragan et al., 2014) to chart which media activities individuals engage in, or during 15-min study sessions to investigate predictors and patterns of media multitasking over time (Rosen et al., 2013). Another observational approach by Voorveld and Viswanathan (2015) involved observing participants over the course of a day to understand what types of televised content are associated with media multitasking over longer periods of time. Related methods have involved experience sampling by either briefly interrupting participants as they complete real-world tasks (e.g., during a lecture; Wammes et al., 2019) or probing individuals at several timepoints over the course of the day (Moreno et al., 2012; Xu & Wang, 2022) to acquire estimates of and motivations for media multitasking in situ.

Observational approaches often successfully capture both core components of media multitasking with regards to media centrality and non-required engagement, in addition to accounting for specificity in how this behaviour progresses over time. However, these methods typically rely on researcher and/or participant reports rather than on an objective measure of media multitasking, making them more complex when study designs need to be scaled up for longer sessions or multiple sites. Moreover, these approaches are also not ideal for observing fine-grained temporal changes in media multitasking, which may be problematic given that the focus of one’s attention can change rapidly on a moment-to-moment basis.

#### Laboratory tasks

Recent laboratory work has attempted to overcome these restrictions by providing participants with the option to media multitask during laboratory tasks while objectively recording instances of this behaviour across time during the task. In one such study, Brasel and Gips (2017) presented participants with televised content and a webpage on a split screen, and used an eye tracker to monitor how participants switched their attention between the two forms of media. This allowed for a detailed examination of how specific media content related to the onset of switches in order to determine what types of content cued media multitasking. To further position media multitasking as a natural component of the task, Ralph and colleagues (2020) pioneered a paradigm in which they allowed participants to turn a video on or off at any time when completing an attention-demanding task (i.e., *n*-back; Kirchner, 1958). This method has since been employed to understand how media multitasking differs as a function of task demand (Ralph et al., 2020) and motivation (Ralph et al., 2021). Along similar lines, Lopez and Orr (2022) presented participants with occasional pop-up notifications during an arithmetic task, signalling an opportunity to switch tasks if desired. These laboratory-based paradigms have allowed researchers to study patterns of media multitasking and the antecedents and effects of this behaviour.

Though all of these laboratory tasks are still in early stages of research, they provide a promising avenue for capturing objective and prototypical measures of media multitasking within a controlled laboratory environment to examine how this behaviour shifts and fluctuates over time.

### The five interactive components that drive media multitasking

**Fig. 1:**
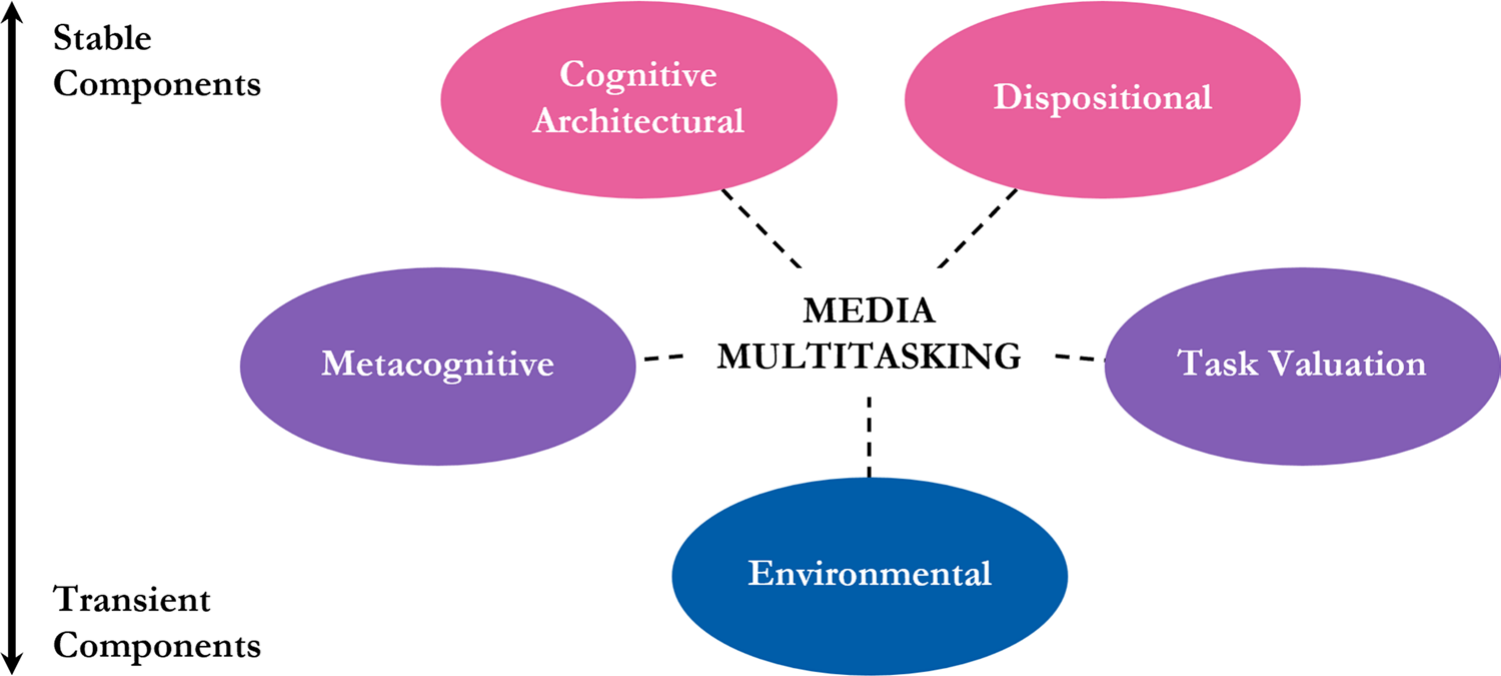
Depiction of the five components within the interactive model of media multitasking, which exist on a core temporal dimension that ranges from stable and intrinsic (above, pink) to transient and mutable (below, blue)

The three aforementioned approaches to studying media multitasking have been fruitful for examining this behaviour across various perspectives and at differing degrees of temporal resolution. This has ranged from the general tendency to engage in media multitasking in daily life (Baumgartner et al., 2017; Ophir et al., 2009) to patterns of media multitasking over the course of a day or across several days (Moreno et al., 2012; Voorveld & Viswanathan, 2015), to fluctuations in media multitasking within a single laboratory task (Brasel & Gips, 2017; Drody et al., 2023; Lopez & Orr, 2022; Ralph et al., 2020, 2021). The findings that have emerged indicate that above and beyond the relatively static factors that drive dual-tasking (e.g., executive functioning difficulties, impulsive traits) are varying factors that change how and when individuals media multitask over shorter time scales (e.g., needs, motivation). The presence of static and varying factors implies that a comprehensive interactive model which captures a range of *stable components* (reflecting unchanging and fixed capabilities and characteristics) to *transient components* (reflecting variable and shifting considerations and contexts) would be ideally suited to uncovering how and why media multitasking manifests over time. Below, and as outlined in Fig. 1, we define and describe these five interactive components and broadly situate various media multitasking findings within each.

#### Cognitive architectural component

The first component reflects primarily stable neural and cognitive features within individuals that place structural and processing limitations on the degree to which one engages in media multitasking. That is, certain individuals may have the capacity to successfully allocate their resources across multiple tasks, whereas others may be unable to do so or may do so inadequately. Support for the role of a cognitive architectural component can be seen through previous research demonstrating that media multitasking is higher among individuals with poor cognitive control (Matthews et al., 2022; Ophir et al., 2009) or tendencies that reflect poor cognitive control, such as higher distractibility (Moisala et al., 2016), lower impulse control (Toyama & Hayashi, 2022), poorer decision-making (Müller et al., 2021), and greater executive difficulties (Magen, 2017; Rogobete et al., 2020). Complementing these findings, research also shows that media multitasking decreases with age (Carrier et al., 2009; Matthews et al., 2022; Voorveld et al., 2014), which may reflect more efficient allocation and deployment of resources for younger versus older individuals (Clapp et al., 2011; Prakash et al., 2009). Studies have also found that media multitasking is more common in women than men (Duff et al., 2014; Jeong & Fishbein, 2007; Kononova, 2013; Lui et al., 2021), likely due to gendered differences across norms, technology usage, and engagement (Cotten et al., 2014; Foehr, 2006; Lucas & Sherry, 2004), but also potentially reflecting gendered differences in executive functioning and spatial coordination abilities (Kimura, 1999; Mäntylä, 2013; Strayer & Ward, 2013). In this manner, each individual’s cognitive architectural component influences media multitasking by placing constraints on the degree to which they can maximize and optimize their limited resources versus overwhelming them.

#### Dispositional component

 The second component reflects relatively stable features within individuals that speak to personality characteristics and trait-level differences that make one more or less prone to media multitask. For example, across personality dimensions, prior work has demonstrated that individuals high in conscientiousness media multitask more (in specific contexts; Toyama & Hayashi, 2022, 2023). Studies have also provided support for links between media multitasking and both negative affect (Becker et al., 2013) and positive affect (Hatchel et al., 2018) depending on individual differences in factors such as social anxiety and narcissism (Hatchel et al., 2018). Similarly, across traits, media multitasking is found to be higher for individuals who are poor in time management (Yang & Zhu, 2016; Yang et al., 2015) and high in impulsivity (Magen, 2017; Minear et al., 2013; Sanbonmatsu et al., 2013; Schutten et al., 2017), sensation-seeking (Y. Chang, 2017; Duff et al., 2014; A. Kononova, 2013; Lim & Shim, 2016; Sanbonmatsu et al., 2013), and boredom proneness (Drody et al., 2022; Nagar & Singh, 2020). Additional evidence can be seen when examining overall mood, wherein studies have established connections between media multitasking and decreased feelings of normalcy (Pea et al., 2012), decreased self-esteem, and greater depression and social anxiety (Becker et al., 2013). In this manner, each individual’s dispositional component acts to enhance or hinder their ability to focus on and prioritize a single task, playing a strong role in their general tendency to engage in media multitasking.

#### Metacognitive component

The third component reflects features that are both stable and transient and pertains to the degree to which an individual’s awareness of themselves, in terms of their ability to media multitask effectively, along with their confidence and error detection in this ability, can influence their decision to engage in this behaviour. These forms of metacognitive beliefs are typically known to be stable (Cartwright-Hatton & Wells, 1997; Faissner et al., 2018; Nordahl et al., 2023), such that they are gradually formed over time as individuals accumulate more experiences with a particular behaviour (Bandura, 1977; Rouault & Fleming, 2020); however, there is a strong transient component to aspects of self-monitoring wherein the presence of new information can quickly alter a person’s perception of their beliefs (Gilbert et al., 2019; Grinschgl et al., 2021; O’Leary & Fletcher, 2024).

Within the media multitasking literature, there appears to be large variability in findings regarding individuals’ understanding of the consequences of their media multitasking behaviours. On the one hand, studies have shown that individuals can accurately perceive their own metacognition about media multitasking, anticipating the extent to which media multitasking will hinder upcoming performance (Froese et al., 2010; Gingerich & Lineweaver, 2014), retrospectively judging the degree that this behaviour impacted their prior performance (Gingerich & Lineweaver, 2014; Ralph et al., 2020), and varying media multitasking behaviours for tasks that are low versus high demand when performance is likely to be impacted (Baumgartner & Wiradhany, 2022; Ralph et al., 2020; Wang et al., 2015). On the other hand, some studies have demonstrated that individuals are not always capable of anticipating the consequences of media multitasking. For instance, Clayson and Haley (2013) demonstrated that students were incorrect in their belief that they could attend to lectures while texting, whereas Calderwood and colleagues (2016) found that, although participants could accurately predict that media multitasking would impede their ability to complete an assignment, they overestimated the extent to which this behaviour would reduce their mood and ability to stay focused on their task. In this way, each individual’s metacognitive component allows them to use their own understanding of their abilities, whether correct or incorrect, to help them better estimate the degree to which they will or will not experience overall impairments when media multitasking.

#### Task valuation component

The fourth component reflects features that can be either inherently stable or dynamically transient and highlights the value that one places on the tasks and activities that they are currently engaged in over the costs and benefits they attribute to media multitasking. In some cases, task valuation may present as a more stable judgement, broadly devaluing or enhancing one’s general propensity to media multitask. For instance, individuals holistically perceive several global downsides to media multitasking (e.g., poorer task performance, reduced efficiency, negative emotions, feelings of disengagement; Bardhi et al., 2010; Ralph et al., 2020), which would diminish the overall value of this behaviour. However, media multitasking is also chosen because it satisfies various motivational purposes, such as entertainment (e.g., listening to music, browsing the internet), socialization (e.g., texting, use of social media), and information-seeking (e.g., looking up an unknown term during a lecture; Hwang et al., 2014; Wang & Tchernev, 2012), which can easily drive up the overall value of this behaviour.

In addition to these stable judgements, transient influence can also play a role in one’s in-the-moment media multitasking behaviours. For example, work by Kononova and colleagues (2015, 2017), showed that media multitasking can be dictated by an individual’s immediate needs, such as emotional (i.e., improving mood), social (i.e., connecting with others), cognitive (i.e., acquiring knowledge), and habitual (i.e., routine behaviours). Relatedly, laboratory studies have shown that in-the-moment boredom is tied to increases in media multitasking (Drody et al., 2023) and increased in-the-moment task motivation can reduce media multitasking (Ralph et al., 2021). These transient judgements may also influence moment-to-moment temporal changes in rates of media multitasking based on differential valuations (Calderwood et al., 2014; Drody et al., 2023; Ragan et al., 2014; Wammes et al., 2019) and the gratification of these needs over time (Wang & Tchernev, 2012). Together, these studies point to the notion that one’s likelihood of media multitasking may be a result of them weighing the stable and transient rewards of engaging in this behaviour (e.g., fulfilling social and emotional needs) against the multitude of consequences present (e.g., reduced efficiency and engagement). In this way, each individual’s task valuation component allows their media multitasking behaviours to dynamically change and vary over time based on the factors that they consider to be relevant and important at that specific moment.

#### Environmental component

 The fifth component is predominantly transient and emphasizes the role of one’s current environment in media multitasking, which includes the current availability of tasks within any given setting. For example, media multitasking behaviours may be more voluntarily chosen while at home versus at work given the increased opportunities available in the former. In line with this notion, prior work has demonstrated that a strong predictor of media multitasking is the possession of or access to media options (Kononova, 2013; Kononova & Chiang, 2015; Srivastava et al., 2016; Yang & Zhu, 2016), with a majority of participants choosing to media multitask when given the opportunity to do so (Lopez & Orr, 2022; Ralph et al., 2020). In addition, several studies have determined that certain task features are associated with greater media multitasking. For instance, individuals are most likely to media multitask when the tasks involved do not overlap on sensory modality (e.g., having separate audio, visual, or motor contributions) and provide individuals with a high degree of control over the information presented (Baumgartner & Wiradhany, 2022; Wang et al., 2015), allowing them to best adjust how media multitasking presents within their environment. Task demand has also been linked to media multitasking, with this behaviour being more commonly selected during less cognitively demanding tasks (e.g., tasks requiring few responses, Wang et al., 2015; or tasks which place a low demand on working memory, Ralph et al., 2020). Beyond opportunities to media multitask, norms surrounding media multitasking in a given environment may also be a relevant factor, such that individuals are found to media multitask less when they collectively attend to a media stream (Voorveld & Viswanathan, 2015). Moreover, students have cited norms surrounding in-lecture media multitasking as a reason for class engagement with this behaviour (Parry & le Roux, 2017). Together, these findings illustrate that each individual’s environmental component is comprised of various transient features that can influence whether one engages in media multitasking and how they choose to do so.

### Implications of the interactive model

#### Bridging diverse fields of work

In capturing these five components within our interactive model of media multitasking, this work prioritizes cognition at both a group and an individual level, thereby bridging complementary fields of research that have crafted models for understanding human behaviour. Models within the field of performance psychology align with our own in that they predict an influence of transient (i.e., environmental and task valuative) factors on task selection. For instance, the Yerkes-Dodson model (Ma et al., 2017; Watters et al., 1997; Yerkes & Dodson, 1908) implies that tasks or task combinations that promote an optimal level of arousal may be preferred by individuals. Additionally, models emphasizing cost-benefit trade-offs, such as the opportunity costs model of mental effort, suggest that experiential factors like boredom and fatigue promote task-switching (Kurzban et al., 2013).

Decision-making frameworks also emphasize transient influences on choice behaviour, which likely extend to one’s decision to media multitask. For instance, contingent weighting models (Tversky et al., 1988) propose that the value individuals assign to different choice attributes when making a decision (e.g., the enjoyment vs. risks associated with single tasking compared to multitasking) varies based on contextual factors, such as the importance of optimal performance in a given setting. Prospect theory (Camerer, 1998) highlights similar influences while emphasizing an aversion to loss when weighing the trade-offs of a decision. Moreover, priority heuristic models (Cokely & Kelley, 2009) further suggest that individual differences in cognitive abilities affect risk assessment during decision-making, underscoring the need for a model that integrates both stable and transient factors. Research in the field of neuroeconomics provides additional evidence for this perspective, identifying prefrontal regions (e.g., orbitofrontal cortex; Lee & Seo, 2007; Lee & Seo, 2007; Murray et al., 2007; Murray et al., 2007) and dopaminergic circuits (Balleine, 2007) involved in predicting the reward value of different courses of action and adapting these predictions to specific situations. By suggesting that cognitive architectural and environmental components shape the value we assign to different choices, this work further supports the necessity of a model that integrates stable and transient influences on media multitasking.

#### Broadening how we characterize media multitasking

 In addition to integrating various models of human behaviour, our interactive model can accommodate diverse theoretical approaches to media multitasking. Many traditional perspectives on media multitasking have considered this behaviour to be harmful, representing a failure of attention and/or self-regulation (Aagaard, 2018; Biedermann et al., 2021; Kokoç, 2021; le Roux & Parry, 2021; Wu, 2017a, 2017b). However, the current model aligns with both negative and positive perspectives on the antecedents and consequences of media multitasking. For instance, our model accounts for how environmental factors, including design features like frequent reminders and auditory/visual notifications, may encourage individuals to media multitask even when this behaviour is likely to distract them from completing important tasks. At the same time, our model allows for the possibility that dispositional and task valuation factors, such as extraversion and a desire to socialize, respectively, may increase one’s likelihood of media multitasking, potentially leading to positive outcomes. For example, media multitasking with the goal of socializing could help strengthen one’s social connections, thereby improving their overall well-being (Xu et al., 2021, 2022). In this way, our model aligns with recent theories of media multitasking which take a more neutral stance on this behaviour rather than emphasizing its costs alone (e.g., Wiradhany et al., 2021; Xu et al., 2022).

#### Capturing variability in media multitasking behaviours

**Fig. 2:**
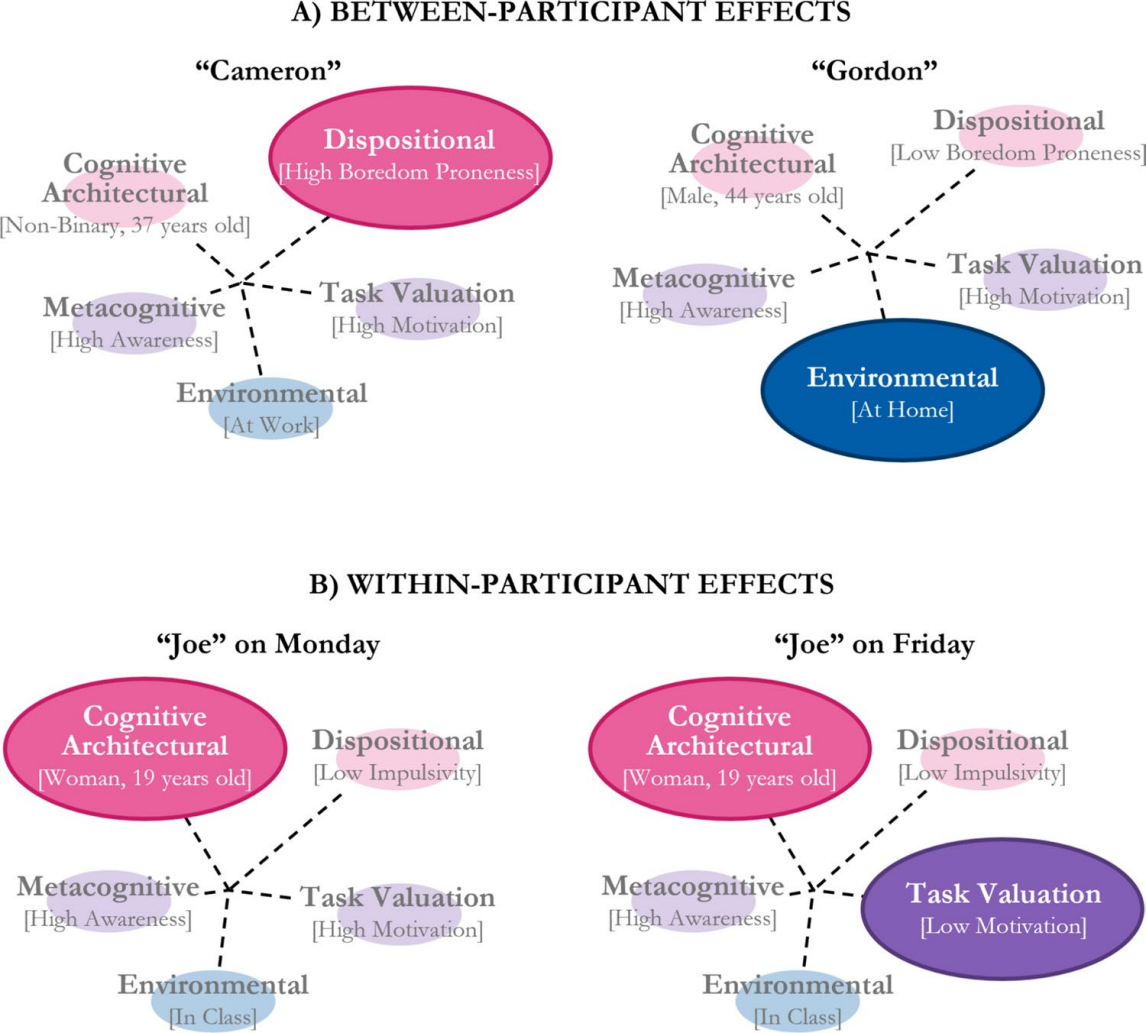
Graphical depiction of a hypothetical scenario in which (**A**) two individuals with different levels of boredom proneness show a similar likelihood of media multitasking due to differences within the environmental component, and (**B**) the same individual has a different likelihood of media multitasking over consecutive days due to variability within the task valuation component. In both figures, the size and opacity of the ovals represent the degree to which each component is expected to impact one’s decision to media multitask

Beyond this neutrality, our interactive model allows us to account for the complex and variable patterns of media multitasking that individuals display on a daily basis. Critically, the interactive model can explain why individuals with different stable components or virtually identical transient components may media multitask in ways that are in opposition to previously predicted relationships. Such instances are depicted in Fig. 2, which illustrates hypothetical media multitasking scenarios, with ovals representing each component and the size and opacity of each oval representing the extent to which the component encourages media multitasking. For example, if considering between-group effects, media multitasking may be higher for individuals who have a greater disposition towards this behaviour (e.g., high vs. low boredom proneness); but as seen in Fig. 2A, this difference may not hold if environmental opportunities to media multitask are not abundant (e.g., at work vs. at home). Additionally, if considering within-person effects, an individual put in the same environment on different days (e.g., a student attending a lecture) should have the same cognitive architectural, dispositional, and environmental constraints, and therefore be expected to media multitask at the same overall rate on both days; however as seen in Fig. 2B, media multitasking may differ across days because their motivation to perform well differs between days (thereby changing their task valuation).

#### Understanding the complexity of media multitasking

**Fig. 3:**
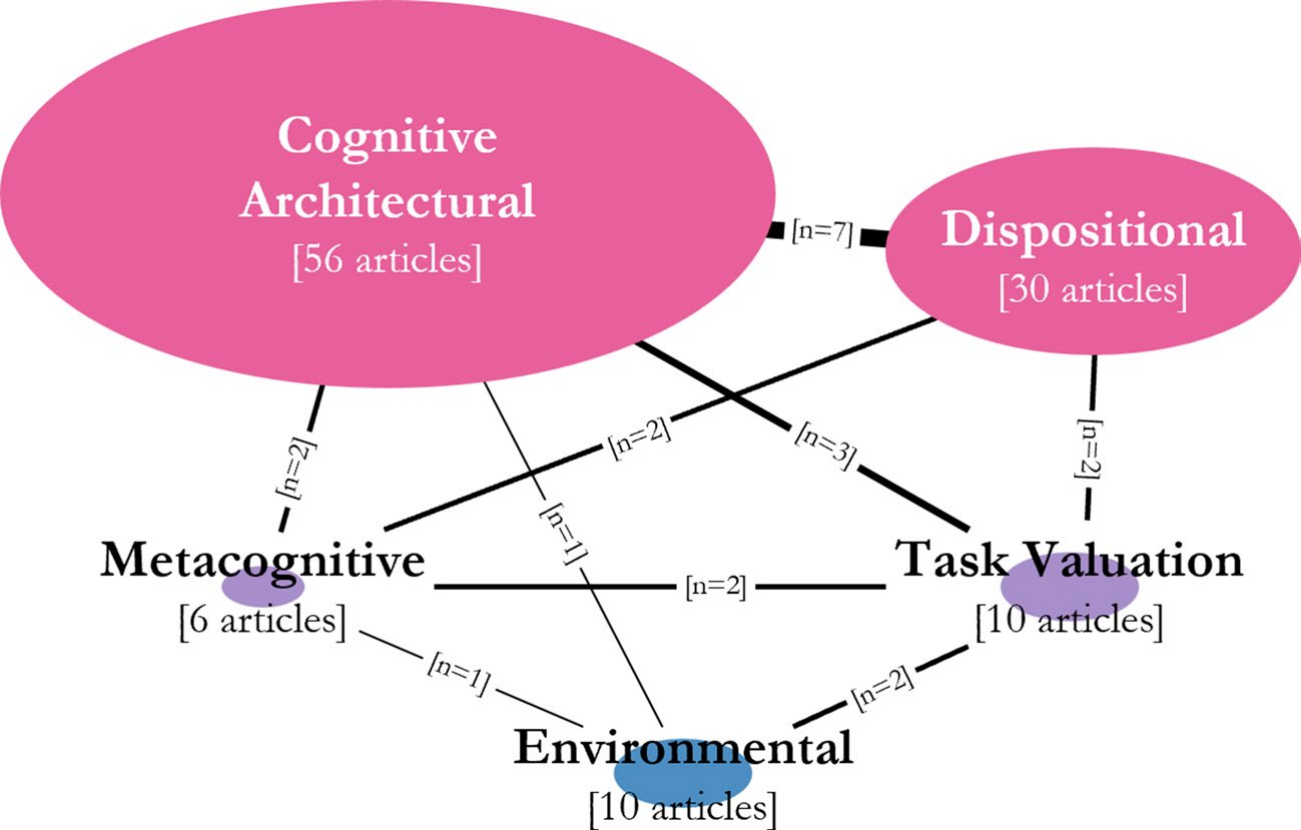
Graphical depiction of existing research on media multitasking that addresses the various interactive components. The size of each oval depicts the number of publications on media multitasking that pertain to the specific component and the width of the lines between each oval depicts the number of publications on media multitasking that have investigated the shared influence between specific components

 Importantly, our model also implies that an accurate, robust, and stable understanding of media multitasking can only occur when each and every component is taken into consideration. Prior work studying either stable or transient components have contributed substantially to our understanding of media multitasking, as seen in Fig. 3, which graphically summarizes the media multitasking research conducted to date both within each component (depicted as the size of the oval) and across components (represented by the width of connecting lines).^1^ However, this sole or primary focus on single components has led to large variance in the range of media multitasking seen within studies (Drody et al., 2023; Ragan et al., 2014; Rosen et al., 2013; Wammes et al., 2019) and inconsistent findings across studies (Kong et al., 2023; Wiradhany & Koerts, 2021; Wiradhany & Nieuwenstein, 2017), implying that additional components may need to be considered for a more complete understanding of this behaviour.

For this reason, our model can help account for many of the inconsistencies observed in the media multitasking literature. For example, it can offer insight into why prior studies employing questionnaire measures of media multitasking have yielded mixed results. Because questionnaires like the MMI do not explicitly account for differences in exposure to transient factors while media multitasking (e.g., environment, task importance), individuals with similar scores on these measures may media multitask in vastly different ways (e.g., during important tasks vs. leisure time). As a result, relations between scores on media multitasking questionnaires and various outcomes may differ across studies depending on the transient factors implicitly captured in each sample. Additionally, studies linking questionnaire measures of media multitasking to individual differences in stable components, such as cognitive control (Kong et al., 2023; Matthews et al., 2022; Ophir et al., 2009; Parry & Le Roux, 2021; Wiradhany & Nieuwenstein, 2017), often measure these components in the laboratory. However, the transient factors present in the laboratory (e.g., the presence of a researcher, the types of distractors used in the task) may differ from those implicitly captured in measures like the MMI, which could further contribute to inconsistencies within the literature. Supporting the importance of accounting for transient factors even when exploring the influence of stable ones, many studies have failed to find relations between the MMI and laboratory measures of media multitasking (Lopez & Orr, 2022; Lui et al., 2023; Shih, 2013; although, see Riby et al., 2017). As such, a potential benefit of studying media multitasking within our interactive model is that it could afford researchers better predictive ability by allowing variability in media multitasking to be distributed across multiple components of the model and across their interactions.

### Implications of the interactive model for future research

Explaining media multitasking behaviours through the lens of interactive components introduces several broad and fruitful avenues for future research.

#### Weighting of components

One major theoretical question concerns the weighting of each component in one’s likelihood of media multitasking. That is, do some components exert a larger influence on media multitasking than others? To exemplify this point, we can consider two possible individuals who vary in their dispositional component (e.g., high vs. low self-regulation), with each having different task valuation components (e.g., high vs. low reward value of the media activity). When considering behaviour, it is plausible that the individual with low self-regulation and high reward valuation is the most likely to media multitask and the individual with high self-regulation and low reward value is the least likely to media multitask. What is less clear however is whether the situation in which both self-regulation and reward value are low is just as likely to lead to media multitasking as when both self-regulation and reward value are high. If rates of media multitasking differ across these scenarios, this would provide insight into whether dispositional or task valuative components (or specific variables within them) are more influential in predicting one’s likelihood of media multitasking.

Building on this, an additional question concerns whether the relative weighting of components differs depending on the nature of one’s media multitasking behaviours, such as whether media multitasking is engaged reactively or deliberately. For example, it has been suggested that individuals high in trait-level self-control tend to engage in *less* rather than more state-level self-control in daily life, in part because they avoid situations that might require them to resist temptations (Hofmann et al., 2012; Inzlicht & Roberts, 2024). It is therefore possible that when predicting reactive instances of media multitasking, stable dispositional factors like trait self-control have little impact relative to more transient factors (e.g., task valuative and environmental); however, when predicting deliberate instances of media multitasking, the weighting of dispositional factors may be comparatively greater. Understanding the nuances in how each component is weighted, as well as how the weighting of components differs across scenarios, will be important for creating a thorough understanding of how and why individuals select for media multitasking behaviours.

#### Interactions between stable and transient components

 Another question that arises from this model concerns the interplay between different components. Given that much of the current research on media multitasking has focused on the influence of either stable or transient components on media multitasking, investigating the interactions between these categories of components would meaningfully advance our understanding of media multitasking. To this end, researchers could examine whether certain stable factors bias individuals’ processing of transient factors. For instance, it is possible that cognitive architectural factors like attention-deficit hyperactivity disorder (ADHD), characterized by tendencies towards inattention, hyperactivity, and impulsivity (American Psychiatric Association, 2013), bias processing within the task valuation component. Specifically, individuals with ADHD may be particularly sensitive to the rapid rewards provided by many digital media platforms (Dekkers & van Hoorn, 2022), increasing the value they assign to engaging with media and ultimately increasing their likelihood of media multitasking. Researchers could also investigate whether long-term exposure to certain transient factors can impact stable factors. For example, there is some evidence to suggest that frequent interruptions from notifications, an environmental factor, might increase dispositional factors such as a fear of missing out (Brown & Kuss, 2020; Przybylski et al., 2013; Rozgonjuk et al., 2019), but to date, no studies have investigated these interactions.

#### Interactions within stable or transient components

 Additionally, researchers could investigate interactions within components by studying how different stable components or various transient components interact and influence one another. An interesting question involving stable components concerns whether cognitive architectural factors like poor decision-making interact with dispositional factors like high impulsivity to give rise to heightened rates of media multitasking. Regarding transient components, researchers could explore whether certain environmental factors, such as the opportunity to switch between reading an article and engaging with one’s phone, influence task valuation factors, such as one’s level of interest in the article. Supporting the interplay of transient components, Tam and Inzlicht (2024) found that boredom motivated individuals to switch media, but that the act of switching also exacerbated feelings of boredom. An intriguing implication of this finding is that the components within our model have the potential to reinforce one another (as in the case of boredom and the opportunity to switch tasks; Tam & Inzlicht, 2024) or counteract one another (e.g., sustained attention difficulties may be offset by motivation to complete a primary task), leading to complex downstream effects on media multitasking behaviours.

#### Interventions for behaviour

The presence of interactive components also allows us to identify interventions that can be effective at reducing media multitasking in daily life. The most salient interventions would likely target transient components as they may be more sensitive to immediate and momentary changes in one’s decision to media multitask. For instance, targeting the metacognitive component might involve making individuals more aware of their ability to effectively media multitask, a notion supported by work in health psychology that suggests that interventions targeted at changing an individuals’ attitudes (e.g., towards health-promoting behaviours) lead to corresponding behavioural changes (Jones et al., 2015; Sheeran et al., 2016). Studies have attempted to modify media multitasking behaviours by educating individuals about its negative impact on task performance (Tassone et al., 2020; Terry et al., 2016); however, these interventions have been unsuccessful to date, possibly because effectively altering beliefs requires long-term management or consideration of other transient components (e.g., the impact of different contexts within the environmental component). Other methods that may target metacognitive variables and have been successful at reducing media use include increasing participants’ awareness of their time spent engaging with distracting media as well as encouraging them to set goals related to reducing their engagement with these forms of media (Biedermann et al., 2021). Targeting the task valuation component could involve tapping into motivational reasons that reduce the discrepancy between the value associated with attending to a singular task versus media multitasking. Increasing task motivation has been effective at reducing individuals’ likelihood of media multitasking in the laboratory (Ralph et al., 2021), implying that avenues focused on incentives and enjoyment (e.g., gamification of assigned tasks; Gerdenitsch et al., 2020; Sailer & Homner, 2020) could prove beneficial. Finally, targeting the environmental component could entail removing or changing the nature of the media multitasking opportunities in one’s environment. Several studies have demonstrated that apps which block media distractions may be effective at reducing media use (Kim et al., 2016, 2019), suggesting that exclusionary strategies might be effective at reducing media multitasking (see Biedermann et al., 2021, and Parry & Le Roux, 2019, for reviews on such interventions).

#### Design of digital technology

 Our interactive model also provides insight into why individuals are so prone to multitasking with current digital media devices by speaking to how features of digital media platforms are designed in ways that tilt the weighting of transient components towards a greater likelihood of media multitasking. For instance, app notifications and digital nudges remind individuals of the opportunity to media multitask (Chang et al., 2023; Wu & Cheng, 2019); self-scrolling features on platforms such as Instagram and TikTok provide frequent rewarding content in the form of entertaining video reels at unpredictable intervals (Anderson & Wood, 2021); and split screen and auto-scroll features may facilitate media multitasking by requiring little action on the part of the user in order to initiate and maintain this behaviour (Wang et al., 2015). These features provide salient examples of how digital media platforms are able to seamlessly and effortlessly co-opt our attention.

In addition to providing a framework for understanding and reducing the power of digital media platforms in media multitasking, these design features can also be leveraged to test predictions within the interactive model in ecologically valid ways. Some studies have already begun examining features that act on the environmental component by manipulating the salience of media distractions, such as by altering the frequency or presence of app notifications, to examine how these features influence users’ attention and distractibility (Fitz et al., 2019; Kushlev et al., 2016; Pielot & Rello, 2017). These approaches could also be used by researchers interested in understanding how these features influence media multitasking in particular. Exploring the task-valuation component could involve contrasting self-scroll versus auto-scroll settings and examining their influence on reward valuation and the decision to media multitask. Using design features of digital technology as a tool to understand media multitasking should allow us to better unpack the interactive ways in which we are subjected to digital forms of attentional capitalism (Wu, 2017a, 2017b).

#### Methods for research

 Although the influence of stable components in one’s decision to media multitask has been extensively studied in the past, the influence of transient components as well as interactions between components are under-represented topics in the literature (see Fig. 3 for reference). Capturing and studying these transient components and/or their interactions will require a more dynamic assessment of media multitasking than what has been studied in the past. As noted, questionnaires can be a useful starting point to measure cognitive architectural and dispositional components, and observational reports captured via experience sampling methods within real world activities (Moreno et al., 2012; Xu & Wang, 2022) can allow us to study transient components on a more fine-grained temporal level. Building on this further, laboratory tasks that can capture media multitasking and active choice without interruption (e.g., via retrospective probing, Pereira et al., 2023; Wammes et al., 2019; or providing the option to media multitask, Lopez & Orr, 2022; Ralph et al., 2020) will further bolster the collection and assessment of high-resolution temporal data, allowing for the use of more complex analyses that can account for individual variability within the interactive model when tracking patterns of media multitasking.

## Conclusions

The prevalence of media distractions in daily life does not appear to be slowing (Segijn et al., 2017; Voorveld et al., 2014), necessitating an understanding of the increasingly common behaviour of media multitasking. In this article, we have argued that a robust conceptualization of media multitasking is only possible once researchers consider dynamic instances of this behaviour when it is not required by the task environment. To this end, we have provided a model for understanding media multitasking within this framework by focusing on five interacting stable and transient components – cognitive architectural, dispositional, metacognitive, task valuation, and environmental – that give rise to this behaviour. Critically, our interactive model has implications for enhancing our ability to predict media multitasking in our real and digital worlds, as well as improving methods of capturing this behaviour and its predictors in real time. Moreover, our model provides instrumental pathways and directions for future research that addresses the ongoing need to decrease instances of media multitasking in contexts in which it can have deleterious effects, and to reduce the reach of digital media platforms which leverage model components in competition for our attentional economy.

## Supplementary Information

Below is the link to the electronic supplementary material.

Supplementary file1 (XLSX 21 KB)

## Data Availability

The data used to create Fig. 3 can be found in the Online Supplementary Materials.

## Footnotes

1 This figure was created based on a PubMed search conducted in May 2024 using the key term “media multitasking,” which returned a total of 250 articles. Articles were then selected for figure inclusion if they explored relations between media multitasking and (i) at least one of the five components of the interactive model or (ii) the joint influence of multiple components of the interactive model (e.g., the interaction between two components when predicting media multitasking or the influence of one component while controlling for others). This resulted in a final set of 72 articles. For each article, we used binary coding (0 = No, 1 = Yes) to indicate whether the article captured one of the five components contained within the interactive model or captured interactions or relations between possible pairs of components (e.g., cognitive architectural and dispositional, task valuation and environmental). Summing each category then provided the respective number of articles examining this topic in relation to media multitasking, with the size of the oval representing the number of papers that examined each component alone and the size of the lines connecting components representing the number of papers that jointly examined components. The data used to create this figure can be found in the online Supplementary Information. Articles that were used to create this figure and were cited in the present article are marked by an asterisk in the reference list.

## Declarations

### Open practices statement

This review was not preregistered. The data used to create Fig. 3 can be found in the Online Supplementary Materials.
